# The Language of Medicine

**Published:** 1888-06

**Authors:** 


### Book Reviews.

**The Language of Medicine.** By F. R. CAMPBELL, A. M., M. D., Professor of Materia Medica and Therapeutics, Medical Department of Niagara University. New York: D. Appleton & Co. 1888.

This is a handsome octavo volume of 325 pages, and has for its object, as the author states in the preface, to provide the medical student with a suitable means of acquiring the vocabulary of his science. In Part I. the origin of the language of medicine is discussed, and includes a history of medicine from a linguistic point of view. The author shows himself thoroughly familiar with the principles of philology, and has succeeded in writing some chapters that will be of great value and interest to the educated physician as well as the novice. Each philological law is illustrated by examples of words in common use in our medical text-books.

In Part II. is found a discussion of the Latin element in the language of medicine. There are chapters on the proper pronunciation of medical words, and a list of 500 words commonly mispronounced, the correct and incorrect pronunciation being in each case indicated. In the composition of this portion of the work, the author evidently had in mind a little work which has attracted great attention among students, "3,000 Words Commonly Mispronounced." Physicians who are obliged to teach—and what medical man is not engaged in teaching?—will find these chapters of great interest. All will be surprised to learn how many words they have been continually mispronouncing. Elementary instruction is given in those principles of Latin grammar which are of use in nomenclature and prescription writing. Vocabularies are given with the etymology and pronunciation of the words. There is also a chapter giving the grammatical construction of the prescription. In Part III. is found "The Greek Element in the Language of Medicine." The method of converting Greek words into Latin and English is described, and an alphabetical list of the stems of words designating the parts and functions of the body is given. In paralle columns with these stems are found the Greek words with derivations, and the Latin and English equivalents. Then, by means of prefixes and suffixes, the author builds up a host of Greek derivatives found in our medical books. There is also a chapter giving the principles of anatomical nomenclature.

In Part IV. are collected the foreign words adopted into medical terminology from the French, German, Italian, Spanish, etc. The whole work is carefully indexed.

We believe that this work supplies a real want in the profession. As Dr. M. B. Anderson, President of Rochester University, says: "The book will be of great interest even to physicians who have had a liberal education, and to the great mass of students ignorant of the classical languages, it will be found almost invaluable. The author's style has a terseness and perspicuity which render it a pleasure to read, while every page displays careful investigation and thorough scholarship."

